# Ambra1 Deficiency Improves Retinal Inflammation in Streptozotocin-Induced Diabetic Mouse Models

**DOI:** 10.7759/cureus.90937

**Published:** 2025-08-25

**Authors:** Takahiro Suzuki, Takehito Sato, Kaori Masuhara, Mizuki Tokusanai, Hisako Akatsuka, Tomohiro Kashikawa

**Affiliations:** 1 Department of Ophthalmology, Tokai University School of Medicine, Kanagawa, JPN; 2 Department of Immunology, Tokai University School of Medicine, Kanagawa, JPN

**Keywords:** ambra1, cell proliferation, cyclin d3, diabetic retinopathy, retinal inflammation

## Abstract

Purpose

To investigate the effects of *Ambra1*, which is involved in cell proliferation control in diabetic retinal inflammation, on the retina.

Materials and methods

*Ambra1*F/FxRosa-Cre-ER2-Tg mice and control *Ambra1*F/F mice were administered tamoxifen to generate mice with tamoxifen-dependent knockout of the *Ambra1* gene in systemic tissues (*Ambra1* conditional knockout, cKO) mice and control mice, respectively. Each mouse was administered streptozotocin (STZ) to induce type 1 diabetes. Following enucleation, immunofluorescence staining was performed, and the neuroretina was excised and cultured in the presence of 5-bromodeoxyuridine (BrdU). Staining for cyclin D3 and glutamine synthetase (GS) was performed to observe S-phase progression, localization, and characteristics. Further, we examined the expression of glial fibrillary acidic protein (GFAP), a marker of astrocytic activation in neural tissues, which is typically elevated in diabetic retinal inflammation.

Results

In cultured retinal tissues, BrdU staining revealed a significant increase in 5-bromodeoxyuridine (BrdU)-positive cells in *Ambra1* cKO mice compared with controls. Immunostaining for cyclin D3 showed significantly elevated expression in *Ambra1* cKO mice compared to controls, regardless of STZ treatment. However, no significant difference in cyclin D3 levels was found between STZ-treated and untreated groups within either genotype. GS staining showed no significant difference between untreated *Ambra1* cKO and control mice. Following STZ administration, GS expression increased in both groups, with the *Ambra1* cKO mice exhibiting significantly higher levels than their STZ-treated controls. Notably, the regional distribution of cyclin D3 expression closely mirrored that of GS, suggesting potential colocalization. GFAP staining showed that STZ treatment significantly increased expression in control mice; however, this increase was suppressed in *Ambra1* cKO mice.

Conclusion

These results indicate that loss of *Ambra1* function promotes the proliferation of Müller glia-derived cells and may contribute to the suppression of retinal inflammation under diabetic conditions. Modulating *Ambra1* activity could potentially serve as a new therapeutic approach for managing inflammation in diabetic retinopathy.

## Introduction

Diabetic retinopathy (DR) is a leading cause of vision loss [1]. Current treatment options include laser photocoagulation [2], vitrectomy [3], and intravitreal administration of anti-vascular endothelial growth factor (VEGF) drugs [4]. However, with the increasing prevalence of diabetes, the number of patients with DR and visual impairment is also on the rise. Amid the growing demand for novel treatment options, therapies targeting neural regeneration and protection, as well as retinal regeneration, are gaining attention for their potential to not only maintain vision but also improve it. During retinal injury, Müller glial cells within the retina dedifferentiate into intrinsic stem cells (retinal progenitor cells) [5] that further differentiate into retinal neurons [6]. Understanding the mechanisms underlying Müller glial cell reprogramming is key to harnessing the regenerative potential of the retina.

In adult zebrafish, Müller glial cells proliferate following retinal injury and differentiate into major cell types, such as cone cells, rod cells, and optic nerve cells, thereby regenerating the retina [7]. Likewise, in post-hatch chickens, retinal injury stimulates proliferation of Müller glial cells, which exhibit a limited capacity for neurogenesis and give rise to a small number of retinal interneurons [8]. In adult newts, retinal pigment epithelial cells play a crucial role in the regeneration of the entire retina [9]. Conversely, in mammalian retinal injury, although Müller cells support remodeling of neural processes and synapses, abnormal tissue repair (known as gliosis) occurs without the recovery of lost neurons [10].

Focusing on *Ambra1*, an essential gene for proper neural development [11], we successfully created *Ambra1* flox mice using an optimized CRISPR/Cas9 method [12]. We crossed them with Rosa-Cre-ERT2-Tg mice to obtain induced conditional knockout (cKO) mice in which *Ambra1* was knocked out throughout the body by administering tamoxifen after birth. Further investigation using *Ambra1* cKO mice demonstrated that *Ambra1* serves as a key regulator of autophagy, cellular proliferation, and metabolism [13]. In addition to its established role in autophagy, *Ambra1* also functions as an E3 ubiquitin ligase, mediating the degradation of cyclin D1, D2, and D3-critical proteins involved in cell cycle regulation [14-16]. Furthermore, cyclin D3 is reportedly involved in the proliferation and activation of Müller glial cells, as well as gliosis associated with retinal damage [17, 18].

This study aimed to explore the role of *Ambra1* in diabetic retinal inflammation using pharmacologically inducible *Ambra1* knockout mice. Given the pivotal role of *Ambra1* in cell cycle regulation, we aim to elucidate its impact on the retina and identify the underlying mechanisms involved.

## Materials and methods

**Figure 1:**
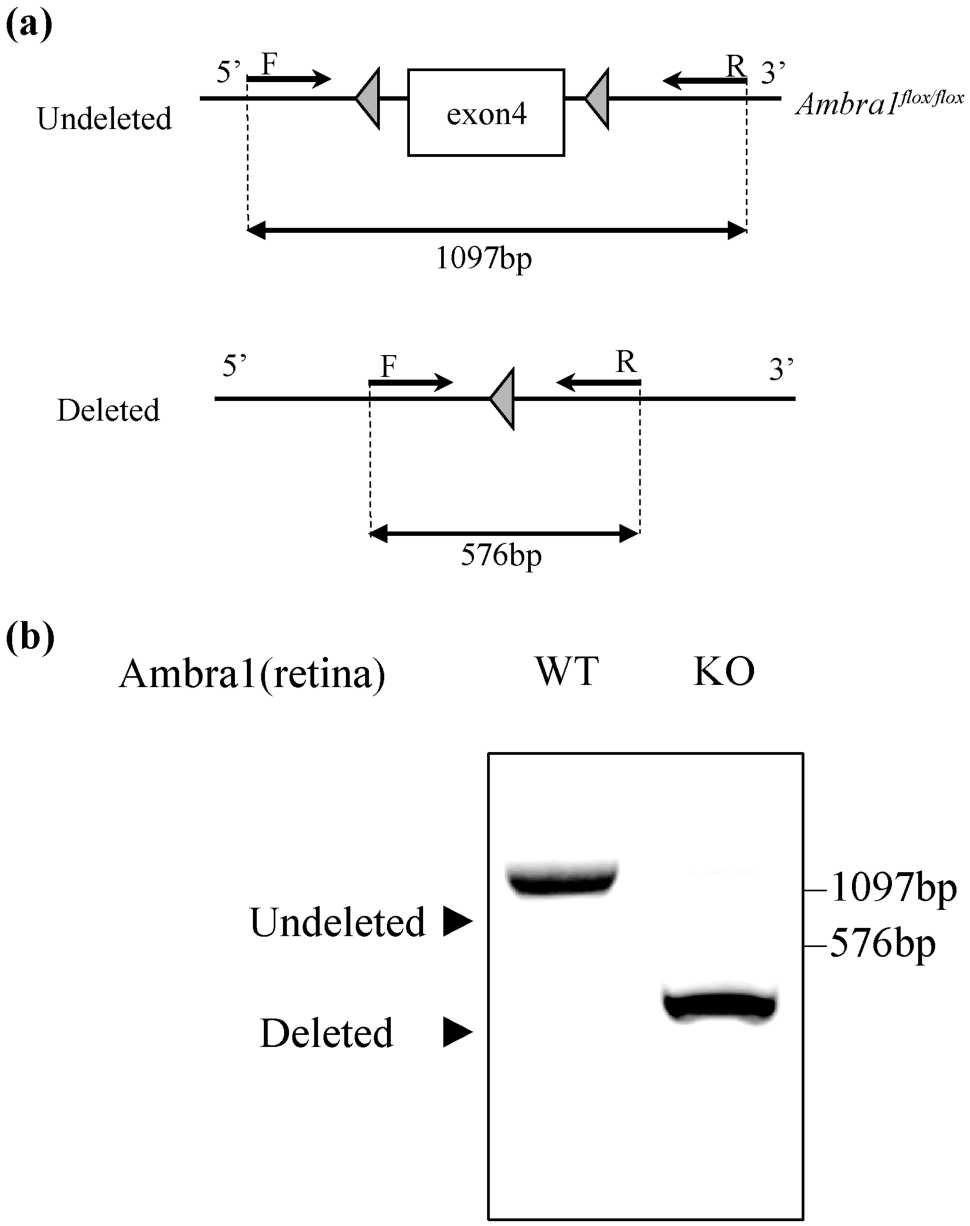
Ambra1 conditional knockout mouse Loss of *Ambra1* expression in AMBRA1 flox/flox x Rosa-Cre-ERT2-Tg mice. (A) Conditional knockout (*Ambra1*-cKO) mice were generated by crossing Ambra1 flox/flox mice with Rosa-Cre-ERT2 transgenic mice, allowing tamoxifen-inducible deletion of *Ambra1*. (B) PCR analysis demonstrated the presence of the *Ambra1 *band in WT retinas, while no such band was observed in KO retinas, confirming effective Cre-mediated deletion of the gene. Abbreviations: WT, wild type; KO, knockout

The Animal Care and Use Committee of Tokai University approved all animal procedures (approval number: 192067; February 19, 2020). To generate inducible systemic *Ambra1* knockout mice, *AMBRA1* flox/flox mice were crossed with Rosa-Cre-ERT2-Tg mice. Both the resulting experimental group and *AMBRA1* flox/flox controls received tamoxifen treatment, and successful *Ambra1* deletion in the retina was verified (Figure 1). Mice were housed under specific-pathogen-free conditions in a temperature-controlled room with a 12-h light/dark cycle and had *ad libitum* access to food and water. Type 1 diabetes was induced by administering intraperitoneal injections of streptozotocin (STZ, 100 mg/kg) [19] on two occasions. One week later, random blood glucose levels were measured from tail vein samples. Mice with glucose levels >300 mg/dL were considered diabetic. Ten weeks after the onset of diabetes, eyes and retinas were collected from diabetic animals for further analysis.

Retinal organ culture

Eyeballs were collected from *AMBRA1 *flox/flox x Rosa-Cre-ERT2-Tg mice that had not been treated with tamoxifen, as well as from *AMBRA1 *flox/flox control mice. To isolate the neural retina, the cornea, sclera, lens, iris, ciliary body, and retinal pigment epithelium were carefully removed. The extracted retinas were placed on microporous Millipore membranes (Merck Millipore, Burlington, USA) and transferred into 6-well culture plates containing medium. Cultures were maintained at 37°C in a humidified atmosphere with 5% CO₂. The culture medium comprised Dulbecco’s modified Eagle’s medium supplemented with 10% fetal bovine serum, 200 μM L-glutamine, and antibiotics. Where appropriate, Wnt3a was added at concentrations ranging from 0 to 100 ng/mL. All key comparisons were performed between *Ambra1*-deficient and control retinas under identical conditions, irrespective of the presence or absence of Wnt3a. To induce retina-specific *Ambra1* deletion, 4-hydroxytamoxifen was added at the initiation of culture. Retinal tissues were collected 5 days after culture initiation, with 5-bromodeoxyuridine (BrdU) added to the medium 24 hours before tissue harvesting. Samples were then subjected to multiplex immunofluorescence staining using a panel of antibodies and analyzed via fluorescence and confocal laser microscopy.

Immunocytochemistry

*Ambra1* knockout mice, their control counterparts, and four corresponding diabetic mouse groups were anesthetized using 3% isoflurane, followed by thoracotomy. To eliminate circulating blood, the left ventricle was perfused with physiological saline, followed by 4% paraformaldehyde (PFA) for fixation via cardiac perfusion. Following enucleation, the cornea, sclera, lens, iris, ciliary body, and retinal pigment epithelium were dissected to isolate the neural retina. The dissected neural retina was immersed in 4% PFA and fixed overnight at 4°C, followed by two washes with phosphate-buffered saline (PBS). Subsequently, retinas were embedded in optimal cutting temperature (OCT) compound (Tissue-Tek, Sakura Finetek, Tokyo, Japan) and stored at −80°C until further processing. Frozen sections (10 μm thick) were prepared using a cryostat (HM505, Microm, Walldorf, Germany). Sections were blocked with 10% normal goat serum at 22 ± 2 °C for 10 min and incubated overnight at 4°C with primary antibodies targeting glial fibrillary acidic protein (GFAP, 1:200; abcam ab7260), glutamine synthetase (GS, 1:100; abcam ab49873), and cyclin D3 (1:20; Santa Cruz sc-6283). After PBS washes, sections were incubated with Alexa Fluor 488-conjugated anti-rabbit IgG (1:400; Thermo Fisher Scientific, Waltham, USA) for two hours at room temperature. Nuclear staining was performed using 4',6-diamidino-2-phenylindole (DAPI, 1 μg/mL) for 15 min at room temperature. Slides were washed five times in 0.5% PBS-Tween and coverslipped using mounting medium (containing DABCO (D27802; Sigma-Aldrich, St. Louis, USA). Immunofluorescent images were captured using a Zeiss LSM 800 confocal microscope (Carl Zeiss, Berlin, Germany).

Organotypically cultured neural retinas were fixed with 4% PFA, rinsed in PBS, embedded in OCT medium, and kept at -80°C following standard procedures. Frozen sections (10 μm) were blocked with 10% normal goat serum and incubated with anti-BrdU antibody (1:100; BD Pharmingen 555627). After PBS washes, sections were incubated with Alexa Fluor 488-labeled anti-rabbit IgG (1:400; Thermo Fisher Scientific) for two hours at room temperature. DAPI (1 μg/mL) was used for nuclear staining, followed by PBS-Tween washes and mounting with Vectashield. Imaging was performed under identical conditions using the same confocal system.

Statistical analysis

Data are presented as the mean ± standard deviation. Statistical comparisons among the four groups were conducted using one-way analysis of variance. When significant differences were detected, Tukey’s post hoc test was applied for multiple comparisons. A p-value <0.05 was considered statistically significant. Statistical analyses were performed using IBM SPSS Statistics version 28 (IBM Corp., Armonk, NY, USA).

## Results

Enhanced proliferative capacity in excised retinas of *Ambra1*-deficient mice

Retinal extraction was performed to induce mechanical damage to the retina, followed by organ culture using the extracted retina. Immunohistochemical staining was performed using BrdU, a marker of DNA replication, to evaluate cell proliferation.

**Figure 2:**
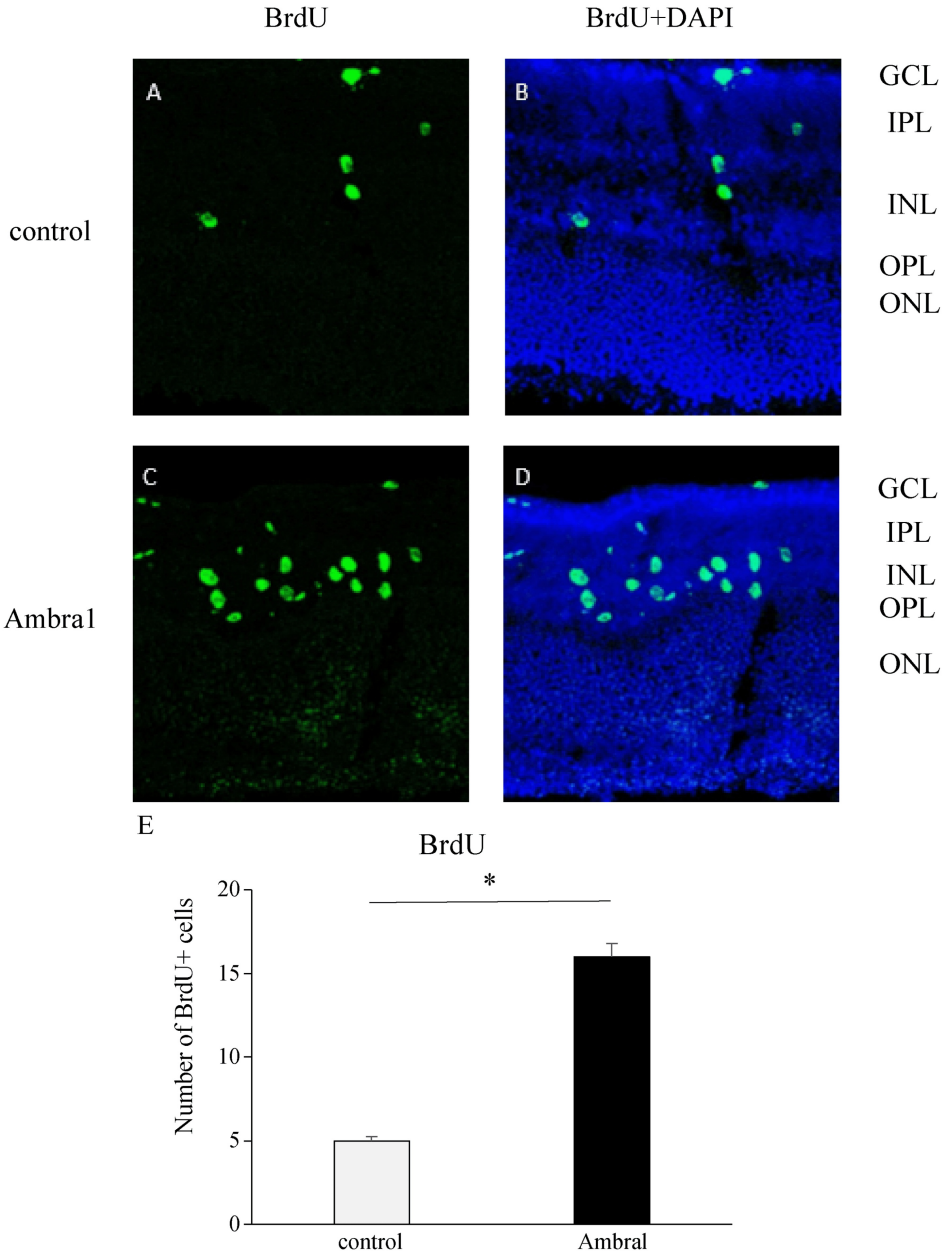
Increased cell proliferation in excised retinas of Ambra1-deficient mice. Retinal extraction was performed to induce mechanical damage, and the extracted retina was used for in vitro culture. Cell proliferation was evaluated using immunohistochemical staining with BrdU, a marker of DNA synthesis. In control mice, BrdU-positive cells (green) were primarily localized in the INL (A, B). In *Ambra1*-deficient mice, a significant increase in BrdU-positive cells was observed within the INL compared with controls (C, D). Quantitative analysis confirmed a significantly higher number of BrdU-labeled cells in the *Ambra1*-deficient group (E). *P < 0.05 by one-way ANOVA with Tukey’s post hoc test. Nuclei were counterstained with DAPI (blue). Abbreviations: BrdU, 5-bromodeoxyuridine; GCL, ganglion cell layer; IPL, inner plexiform layer; INL, inner nuclear layer; OPL, outer plexiform layer; ONL, outer nuclear layer; DAPI, 4',6-diamidino-2-phenylindole.

BrdU-positive cells were predominantly localized in the inner nuclear layer (INL) (Figure 2A-2B). *Ambra1*-deficient mice exhibited a marked increase in BrdU-labeled cells compared with controls (Figure 2C-2D), indicating elevated proliferative activity. Quantitative assessment confirmed a significantly higher number of BrdU-positive cells in *Ambra1*-deficient mice than in controls (P < 0.05, Figure 2E).

Enhanced cyclin D3 expression and GS-derived cell proliferation in diabetic damaged retinas of *Ambra1*-deficient mice

Retinal sections from four groups, including *Ambra1* knockout and control mice, with and without STZ-induced diabetes, were subjected to immunohistochemical analysis. Staining was performed targeting cyclin D3, a central regulator of the cell cycle, and GS, a marker of Müller glial cells.

**Figure 3:**
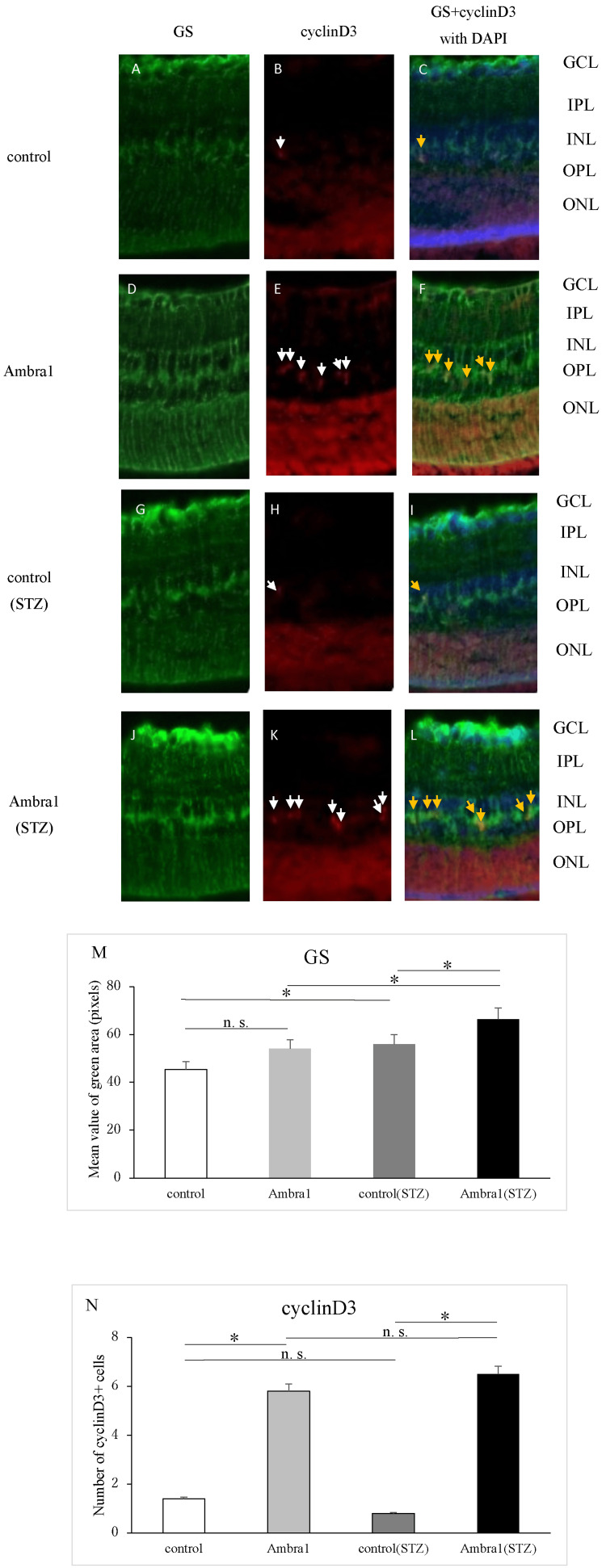
Ambra1 deficiency induces cyclin D3 overexpression and an increase in GS-positive Müller glial cells in diabetic damaged retina. Retinal immunofluorescence staining was conducted on four groups of mice: *Ambra1*-deficient and control mice, with or without STZ-induced type 1 diabetes. In non-diabetic conditions, cyclin D3 (red) was scarcely detectable in control retinas (B, white arrow), whereas *Ambra1*-deficient mice exhibited strong, band-like cyclin D3 expression in the central region of the INL (E, white arrow). Glutamine synthetase (GS; green) was broadly expressed from the IPL to the ONL. Although increased dendritic-like GS-positive structures were observed in *Ambra1*-deficient mice, no significant difference in total GS-positive area was observed between groups (A, D, N). Co-localization of cyclin D3 and GS was observed in several regions (C, F, orange arrows). In the diabetic model, *Ambra1*-deficient mice exhibited a significant increase in cyclin D3 expression in the central INL compared with diabetic controls (H, K, white arrows). GS remained broadly expressed from the IPL to the ONL, and both the extent of GS-positive area and the appearance of protrusion-like structures were elevated in *Ambra1*-deficient mice (G, J, N). Co-localization of cyclin D3 and GS was also evident under diabetic conditions (I, L, orange arrows). (M) Quantification of cyclin D3-positive cells showed significantly higher counts in *Ambra1*-deficient mice relative to controls. (N) Quantification of GS-positive area revealed a significant increase in *Ambra1*-deficient mice under diabetic conditions. *P < 0.05 by one-way ANOVA with Tukey’s post hoc test. Nuclei were counterstained with DAPI (blue). Abbreviations: STZ, streptozotocin; GS, glutamine synthetase; GCL, ganglion cell layer; IPL, inner plexiform layer; INL, inner nuclear layer; OPL, outer plexiform layer; ONL, outer nuclear layer; DAPI, 4',6-diamidino-2-phenylindole.

In non-diabetic mice, cyclin D3 expression was minimal in the control group, whereas *Ambra1*-deficient mice exhibited prominent, band-like expression localized to the central INL (Figure 3B, 3E). Quantitative analysis revealed a significant increase in *Ambra1*-deficient mice compared with controls (Figure 3M). GS was broadly distributed from the inner plexiform layer (IPL) to the outer nuclear layer (ONL). Although *Ambra1*-deficient mice exhibited increased dendritic-like extensions, quantitative analysis revealed no significant difference in GS-positive area compared with controls (Figure 3A, 3D, 3N).

Moreover, in many areas of the retina, the distribution of cyclin D3 closely mirrored that of GS, indicating colocalization (Figure 3C, 3F). In diabetic mice, cyclin D3 exhibited a distinct band-like expression in the central INL, with a significantly higher signal observed in *Ambra1*-deficient mice than in diabetic controls (Figure 3H, 3K, 3M). GS was also expressed from the IPL to the ONL; however, unlike in the non-diabetic model, *Ambra1*-deficient mice in the diabetic model demonstrated a significant increase in GS-positive area and dendritic structures (Figure 3G, 3J, 3N). In the diabetic model, colocalization of cyclin D3 and GS was similarly observed (Figure 3I, 3L).

Effects of *Ambra1* deficiency on retinal inflammation in diabetic damaged retinas of mice

**Figure 4:**
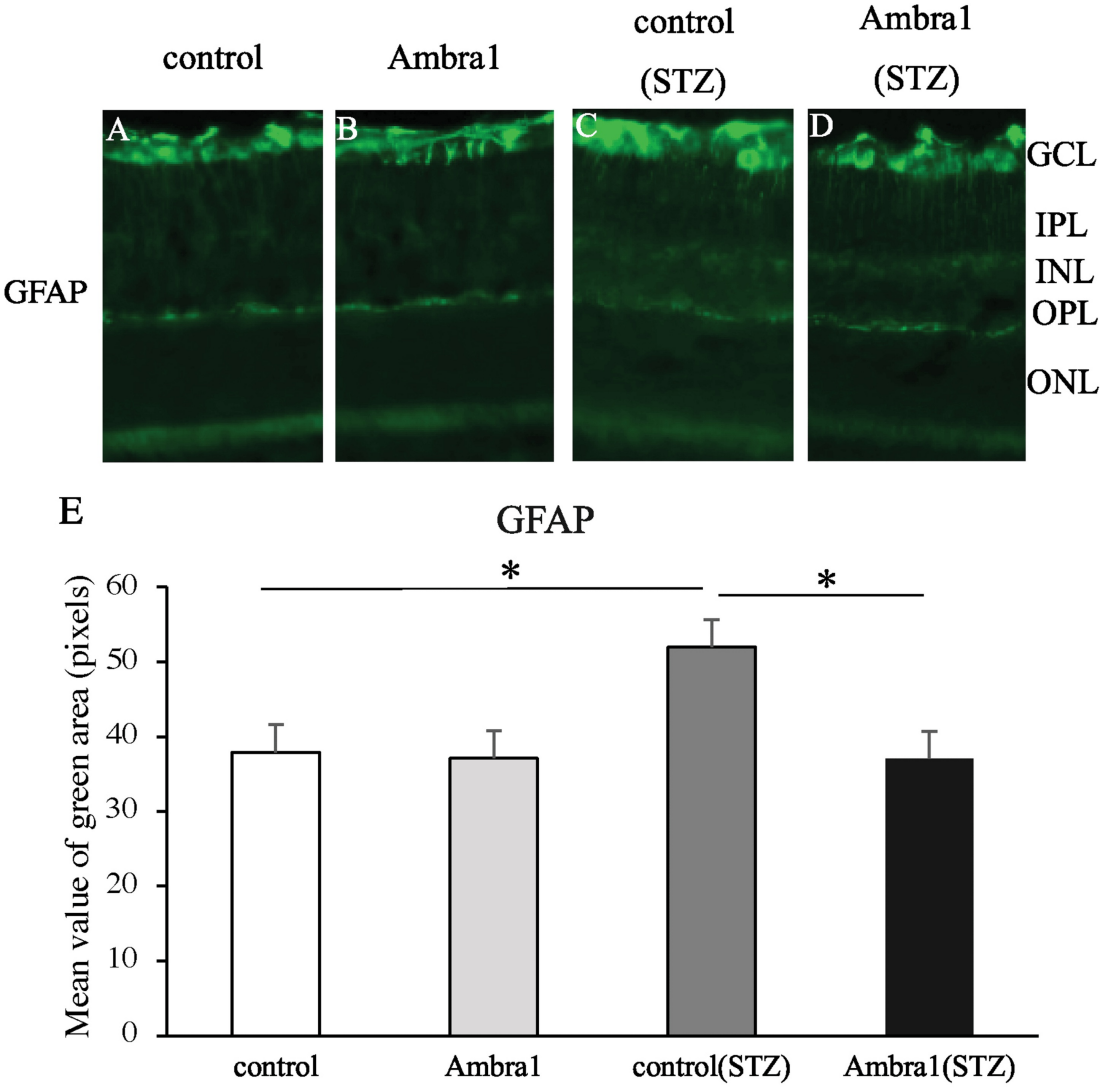
Ambra1 deficiency suppresses the upregulation of GFAP expression in diabetic damaged retina. Immunofluorescence staining for GFAP, a marker of glial activation, was performed on retinal sections from four groups: *Ambra1*-deficient and control mice, with or without STZ-induced type 1 diabetes. In the non-diabetic condition, GFAP immunoreactivity was restricted to the GCL in both *Ambra1*-deficient and control mice, with no significant difference observed between the groups (A, B). In contrast, diabetic control mice exhibited prominent GFAP expression extending from the GCL to the IPL, whereas this upregulation was notably reduced in *Ambra1*-deficient diabetic mice (C, D). (E) Quantitative analysis confirmed a significantly smaller GFAP-positive area in *Ambra1*-deficient mice compared with diabetic controls. *P < 0.05 by one-way ANOVA with Tukey’s post hoc test. Abbreviations: GFAP, glial fibrillary acidic protein; STZ, streptozotocin; GCL, ganglion cell layer; IPL, inner plexiform layer; INL, inner nuclear layer; OPL, outer plexiform layer; ONL, outer nuclear layer.

Immunohistochemical analysis was conducted on retinal sections from four experimental groups: *Ambra1* knockout mice, control mice, and their respective counterparts with streptozotocin-induced type 1 diabetes. GFAP was used as a marker of DR-associated glial activation, and the extent of GFAP immunoreactivity was assessed. In non-diabetic conditions, no significant difference in GFAP-positive area was observed between *Ambra1*-deficient and control mice. However, under diabetic conditions, GFAP expression was markedly elevated from the ganglion cell layer to the IPL in control mice. This increase was significantly attenuated in *Ambra1*-deficient mice compared with diabetic controls (Figure 4A-4E).

## Discussion

In this study, we investigated the role of *Ambra1*, a protein implicated in regulating cell proliferation, in the context of DR. Neural retinas were isolated from both control and *Ambra1*-deficient mice. During isolation, mechanical stress was applied to the tissue, followed by ex vivo culture. Retinas were labeled with BrdU to assess cell proliferation, revealing a significant increase in BrdU-positive cells within the INL of *Ambra1*-deficient retinas compared with controls.

As BrdU is taken up by cells in the DNA synthesis phase (S phase), this increase indicates cell cycle activation (cell proliferation), suggesting that *Ambra1* deficiency facilitates cell proliferation in response to retinal injury, consistent with previous reports [14-16].

Subsequently, we performed immunofluorescence staining for cyclin D3 (a cell cycle-related protein) and GS (a Müllerian glial marker) using eyes from control mice, *Ambra1*-deficient mice, and STZ-induced type 1 diabetes model mice.

In non-diabetic mice, cyclin D3 expression was minimal in the control group but clearly prominent as a band-like pattern in the central INL of *Ambra1*-deficient mice, where it was significantly elevated. A similar distribution pattern was observed in diabetic mice, with cyclin D3 strongly expressed in the central INL and significantly upregulated in *Ambra1*-deficient mice compared with diabetic controls. These findings suggest that the absence of *Ambra1* may disrupt normal cell cycle regulation, potentially facilitating the G1/S phase transition, which is consistent with the increased number of BrdU-positive cells observed. Notably, the enhanced cell proliferation associated with *Ambra1* deficiency was evident irrespective of diabetic status, implying that *Ambra1* may function as a negative regulator of cell cycle progression in retinal tissue. GS expression was broadly observed, spanning from the IPL to the ONL. In the absence of diabetes, *Ambra1*-deficient mice exhibited a trend toward increased GS-positive cellular extensions; however, the difference did not reach statistical significance compared with controls. In contrast, in the diabetic model, GS expression was significantly increased in Ambra1-deficient mice, suggesting enhanced Müller glial cell reactivity.

In diabetic retina, Müller glial cell activation is induced by enhanced inflammatory responses [20]. In this study, cyclin D3 expression was consistently elevated in *Ambra1*-deficient mice regardless of diabetic status, suggesting that the increased GS expression may reflect an inflammatory microenvironment that further promotes Müller glial cell activation.

Furthermore, cyclin D3 and GS were found to co-localize in both diabetic and non-diabetic conditions, indicating that the enhanced cell proliferation associated with *Ambra1* deficiency may originate from Müller glial cells. Immunofluorescence analysis of GFAP, a marker of retinal inflammation, demonstrated a significant upregulation of GFAP expression in diabetic mice compared with non-diabetic controls. However, this elevation was markedly attenuated in *Ambra1*-deficient mice. GFAP is widely recognized as a hallmark of gliosis in Müller glial cells, reflecting a general response to retinal stress or damage [21]. Its upregulation has previously been documented in diabetic models [22]. These findings suggest that *Ambra1* deficiency may contribute to the suppression of diabetes-induced retinal inflammation.

Notably, although GS expression was markedly elevated, GFAP expression was reduced in *Ambra1*-deficient diabetic mice, suggesting that *Ambra1* deficiency enhances Müller glial cell proliferation while concurrently limiting pathological gliosis, thereby contributing to the attenuation of retinal inflammation. Müller glial cells are essential supportive cells within the retina that contribute to its structural stability, homeostasis, and repair processes. They are activated in response to injury and diabetes, exhibiting multifunctional responses such as the secretion of neuroprotective factors and inflammatory cytokines [23]. Furthermore, Müller glial cells can reportedly dedifferentiate into retinal progenitor cells, acting as endogenous stem cells, and subsequently differentiate into neurons [5,6]. However, in the mammalian retina, abnormal gliosis after injury can inhibit nerve regeneration [10], and simply promoting the proliferation of Müller glia may worsen the condition.

In this study, although Müller glia proliferation was observed in *Ambra1*-deficient mice, GFAP expression was suppressed, and inflammation was reduced, without the development of abnormal gliosis.

These findings imply that *Ambra1* deficiency may exert a protective effect against retinal inflammation. One potential explanation is that the enhanced proliferation of retinal progenitor-like cells, originating from Müller glia, contributes to the attenuation of inflammatory responses. In this context, *Ambra1* deficiency may facilitate the dedifferentiation of Müller glial cells into a progenitor-like state, thereby promoting neurogenesis while reducing GFAP expression. Alternatively, *Ambra1* loss may directly inhibit gliotic activation of Müller glial cells. It has been demonstrated that *Ambra1* can downregulate the transcription factor nuclear factor erythroid 2-related factor 2 (NRF2), thereby activating the nuclear factor Kappa B (NF-κB) signaling pathway [24]. NF-κB signaling is activated by retinal injury and is known to promote gliosis, immune cell recruitment, and the suppression of neural progenitor cell dedifferentiation and regeneration [25]. Therefore, disruption or inhibition of *Ambra1* may suppress NF-κB activation, thereby inhibiting glial proliferation and promoting the differentiation of precursor cells with neurogenic potential. We are currently investigating various factors and signaling pathways whose expression is induced or suppressed by *Ambra1* inhibition, including the NF-κB pathway.

Nonetheless, this study has several limitations. First, we employed a whole-body *Ambra1* knockout model, so it remains uncertain whether the observed effects are specific to Müller glial cells. Future research using cell-type-specific genetic manipulation will be essential to clarify this point. Second, we did not assess molecular markers associated with neural progenitor differentiation or retinal regeneration; thus, further studies are needed to determine the role of *Ambra1* deficiency in neuroregenerative processes. Third, while we used the STZ-induced type 1 diabetes model to mimic diabetic retinopathy, this model does not fully replicate the complexity of advanced disease states such as type 2 diabetes or conditions involving neovascularization. Future investigations incorporating alternative models that more closely reflect human pathophysiology, including type 2 diabetes models, are warranted. Finally, to evaluate the translational potential of these findings, validation using human-derived retinal cells or organoid systems will be necessary.

## Conclusions

This study employed *Ambra1* cKO mice to investigate retinal cell proliferation and inflammatory responses in an STZ-induced type 1 diabetes model. Our findings demonstrated that *Ambra1* loss enhanced Müller glial cell proliferation while markedly reducing diabetes-related retinal inflammation, notably without triggering gliotic changes. Although the precise mechanisms remain to be fully understood, the present findings highlight the potential of a new therapeutic approach for DR, one that targets neuroprotection and inflammation modulation, offering an alternative to traditional anti-angiogenic treatments.
